# Phylogenomic Analysis of *Odyssella thessalonicensis* Fortifies the Common Origin of *Rickettsiales*, *Pelagibacter ubique* and *Reclimonas americana* Mitochondrion

**DOI:** 10.1371/journal.pone.0024857

**Published:** 2011-09-21

**Authors:** Kalliopi Georgiades, Mohammed-Amine Madoui, Phuong Le, Catherine Robert, Didier Raoult

**Affiliations:** 1 Unité de Recherche en Maladies Infectieuses Tropicales Emergentes (URMITE), CNRS-IRD UMR 6236, Faculté de la Médecine, Université de la Méditerranée, Marseille, France; 2 Evolutionary Biology and Modeling, LATP UMR CNRS 6632 FR 3098 IFR48, Université de Provence, Marseille, France; Duke University Medical Center, United States of America

## Abstract

**Background:**

The evolution of the *Alphaproteobacteria* and origin of the mitochondria are topics of considerable debate. Most studies have placed the mitochondria ancestor within the *Rickettsiales* order. Ten years ago, the bacterium *Odyssella thessalonicensis* was isolated from *Acanthamoeba spp*., and the 16S rDNA phylogeny placed it within the *Rickettsiales*. Recently, the whole genome of *O. thessalonicensis* has been sequenced, and 16S rDNA phylogeny and more robust and accurate phylogenomic analyses have been performed with 65 highly conserved proteins.

**Methodology/Principal Findings:**

The results suggested that the *O. thessalonicensis* emerged between the *Rickettsiales* and other *Alphaproteobacteria*. The mitochondrial proteins of the *Reclinomonas americana* have been used to locate the phylogenetic position of the mitochondrion ancestor within the *Alphaproteobacteria* tree. Using the K tree score method, nine mitochondrion-encoded proteins, whose phylogenies were congruent with the *Alphaproteobacteria* phylogenomic tree, have been selected and concatenated for Bayesian and Maximum Likelihood phylogenies. The *Reclinomonas americana* mitochondrion is a sister taxon to the free-living bacteria *Candidatus Pelagibacter ubique*, and together, they form a clade that is deeply rooted in the *Rickettsiales* clade.

**Conclusions/Significance:**

The *Reclinomonas americana* mitochondrion phylogenomic study confirmed that mitochondria emerged deeply in the *Rickettsiales* clade and that they are closely related to *Candidatus Pelagibacter ubique*.

## Introduction

*Proteobacteria* are one of the best-studied phyla within bacteria. According to the 16S rDNA phylogeny, *Proteobacteria* are subdivided into five classes: α, β, γ, δ and ε [1]. *Alphaproteobacteria* biodiversity and evolution has been well studied through phylogenetic analyses [2]. Current phylogenomic analysis allows the subdivision of the *Alphaproteobacteria* into six major orders: *Rhodospirillales, Caulobacterales, Sphingomonadales, Rickettsiales, Rhodobacterales* and *Rhizobiales*. Among them, *Sphingomonadales, Rhodobacterales* and *Rhizobiales* have a strong record of free-living organisms and are widespread in aquatic and terrestrial habitats; these organisms also have intracellular lifestyles as plant mutualists or pathogens and animal pathogens [3]. Unlike the three previous orders, *Rickettsiales* members are mostly obligate intracellular bacteria, and either parasitic, for *Rickettsia and Orientia,* or symbiotic for *Wolbachia*. Gene losses often occurred during the evolution of the intracellular species, which explains the small genome sizes of intracellular versus free-living *Alphaproteobacteria* [4]. *Wolbachia* is a special case study that lives in symbiosis with arthropods and annelids. This species shows evidence of genome reduction, but it also experienced several gene integration events from the symbiont genome to the host nuclear genome [5]. It is thought that mitochondria originated through an endosymbiotic event that occurred between the proto-*Rickettsiales* and a pro-eukaryotic cell [6], [7]. Based on biological arguments [8], the endosymbiotic event occurred during the early stages of eukaryotic evolution approximately one billion years ago. Phylogenetic analyses have attempted to reveal the nature of the engulfed bacterium, but this remains a subject of debate [9]. Molecular phylogenomic analyses of whole mitochondrial proteins rooted the mitochondrion among the *Alphaproteobacteria* [10]–[12] but revealed that the heterogeneous origin of mitochondrial genes did not clearly locate the position of the mitochondrion ancestor within the *Alphaproteobacteria* tree. Studies of mitochondrial proteins that are congruent with the *Alphaproteobacteria* evolution place the mitochondrion at the root of the *Rickettsiales* order [13]. *Candidatus Pelagibacter ubique* is a marine free-living bacterium, member of the SAR11 clade, with a small genome and an AT rich genome [14] that was included in the *Rickettsiales* clade since 2007 [2] although there is still discussion on whether a free-living bacterium could be part of a clade including obligate intracellular species [15]. However, phylogenomic studies including *Candidatus Pelagibacter ubique* located the mitochondrion ancestor within the *Rickettsiales* order [2]. Furthermore, its very small and AT rich genome constitute two features that are typical of mitochondria and related obligate intracellular parasites such as the *Rickettsiales* [15]. More recent studies, on the mitochondria of *Chlamydomonas reinhardtii* [10] and *Saccharomyces cerevisiae* [16] find *Rhizobiales* and *Rhodobacterales* as sister taxa of the mitochondria more often that *Rickettsiales*. Therefore, because of limitations in phylogenomic methods and data availability, the origin of the mitochondrial ancestor remains unclear.

Ten years ago, the intra-amoebal gram-negative bacteria, *Odyssella thessalonicensis,* was isolated from *Acanthamoeba* spp. [17]; the 16S rDNA was sequenced and phylogenetic analysis was performed. The resulting tree placed *O. thessalonicensis* in the same clade as *Paraholospora* and in a sister clade to *Rickettsiales*. It was suggested that *Holosporaceae* comprised *O. thessalonicensis, Holospora obtusa, NHP Bacterium* and *Caedibacter caryophilus,* and that it was within the *Rickettsiales* order.

Whole genome shotgun sequencing of *O. thessalonicensis* recently yielded genomic data on a new intracellular *Alphaproteobacteria*. In this study, we have used the sequenced *O. thessalonicensis* genome and the available alphaproteobacterial genomes to reanalyze the phylogenetic position of *O. thessalonicensis* and the evolutionary relationship between the *Alphaproteobacteria* and the *Reclimonas americana* mitochondrion which resembles the most the ancestral proto-mitochondrial genome than any other mitochondrial DNA investigated to date [18].

## Results

### *Alphaproteobacteria* 16S rDNA phylogeny

**Figure 1:**
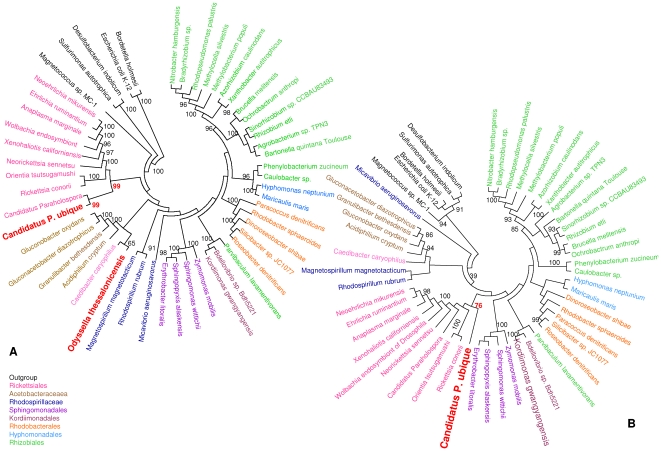
*Alphaproteobacteria* 16S rDNA phylogeny. A. A ML phylogenetic tree of 49 *Alphaproteobacteria* ribosomal DNA sequences is rooted with a non-*Alphaproteobacteria* as outgroup. B. *Alphaproteobacteria* 16S rDNA phylogeny without *Odyssella thessalonicensis*. A ML phylogenetic tree of *Alphaproteobacteria* ribosomal DNA sequences is rooted with a non-*Alphaproteobacteria* as outgroup. Bootstrap values are indicated near branches as a percentage. Different colors correspond to different orders. *Candidatus Pelagibacter ubique* and *Odyssella thessalonicensis* are shown in red.

Phylogenies to recover the position of *Candidatus Pelagibacter ubique* and *O*. *thessalonicensis*, were built with 53 sequences of 16S rDNA, including 49 *Alphaproteobacteria* 16S rDNA sequences, comprising *O. thessalonicensis,* one *Gammaproteobacteria* (*Escherichia coli* K-12), one *Betaproteobacteria* (*Bordetella holmesii*), one *Epsilonproteobacteria* (*Sulfurimonas autotrophica*) and one *Deltaproteobacteria* (*Desulfobacterium indolicum*). Three phylogenetic methods were used: Maximum Likelihood (ML), Maximum Parsimony (MP) and Neighbor Joining (NJ). All three methods yielded the same topology, although branches were better supported by ML and MP methods. The monophyly of *Rhodospirillales* was not supported **(** **Figure 1A** **)**. Instead, this order was split into two clades corresponding to the *Acetobacteraceae* and *Rhodospirillaceae* families. It appears that *O. thessalonicensis* is a sister taxon to the clade formed by *Caedibacter caryophilus* and the four *Acetobacteraceae* species (Bootstrap (BP) = 100). Phylogenies placed *Paraholospora* deep in the *Rickettsiales* clade (BP = 99), while *Candidatus Pelagibacter ubique* is a sister taxon to *Paraholospora*. *Candidatus Pelagibacter ubique* was grouped with *Rickettsiales.* These results were also consistent with those obtained on the phylogenetic tree realized without the *O. thessalonicensis* 16S rDNA sequence **(** **Figure 1B** **)**, however, the topology of *Candidatus Pelagibacter ubique* branching right outside the *Rickettsiales* was not well supported (BP = 76). Both ribosomal DNA phylogenies (with or without *O. thessalonicensis*) also showed that the *Magnetococcus sp*. was the first diverging *Alphaproteobacteria*.

### *Alphaproteobacteria* phylogenomic tree

**Figure 2:**
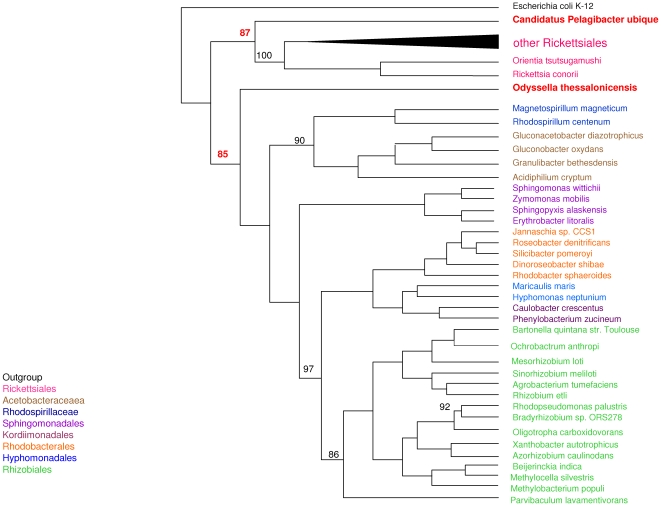
Phylogenomic tree of *Alphaproteobacteria.* Phylogenomic tree of 65 concatenated highly conserved proteins representing the evolution of 42 *Alphaproteobacteria* species. Important bootstrap values are indicated near branches as a percentage. The tree is rooted on *Escherichia coli*. Different orders of *Alphaproteobacteria* are labeled by different colors. *Candidatus Pelagibacter ubique* and *Odyssella thessalonicensis* are shown in red. Some of the *Rickettsiales* species are collapsed.

Because the 16S rDNA does not guarantee an accurate delineation of bacterial species [4], 19,20, we performed a phylogenomic analysis involving highly conserved proteins among 42 *Alphaproteobacteria*. We selected non-duplicated proteins in the *Alphaproteobacteria* proteomes and performed an all-against-all BLAST analysis. Proteins present in all *Alphaproteobacteria* with high-scoring segment pair lengths of more than 150 amino acids and 20% identity were selected; only 65 proteins matched these criteria. The 65 corresponding alignments were performed, conserved blocks were selected, and the resulting cured alignments were concatenated in a single 12,437 amino acid alignment and used for phylogeny construction. The ML and MP methods showed similar topologies with high branch supports, while the NJ method gave very low bootstrap values. The *O. thessalonicensis* clustered together with *Alphaproteobacteria* other than the *Rickettsiales* clades, with high support values (BP = 85), even though the absence of *Holosporaceae* from the dataset does not allow a strong confirmation of this topology **(** **Figure 2** **)**. *Candidatus Pelagibacter ubique* topology as sister taxon to *Rickettsiales* however, was confirmed, as it formed a deep branch alongside *Rickettsiales* also with high support values (BP = 87), and there was an early divergence between the intracellular *Rickettsiales* and the free-living *Pelagibacter* **(** **Figure 2** **)**. The phylogenomic tree suggested that all *Alphaproteobacteria* have evolved from an ancestor located between the *Rickettsiales* clade and the other *Alphaproteobacteria*.

### Mitochondrion and *Alphaproteobacteria* relationship

**Figure 3:**
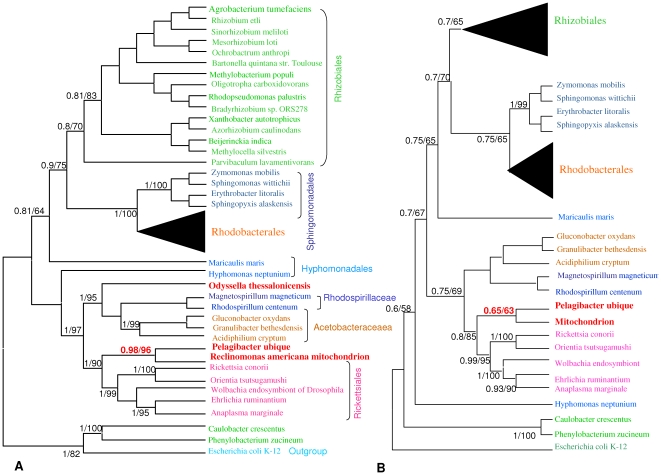
Phylogenomic tree of *Alphaproteobacteria* and the *Reclinomonas americana* mitochondrion. A. ML and Bayesian tree of nine concatenated proteins whose phylogeny is closest to the *Alphaproteobacteria* phylogenomic tree. The *Rhodobacterales* are collapsed. B. Phylogenomic tree of 42 *Alphaproteobacteria* and the *Reclinomonas americana* mitochondrion without *Odyssella thessalonicensis*. The *Rhizobiales* and *Rhodobacterales* are collapsed. Both trees are rooted on *Escherichia coli*. Values near nodes are Bayesian posterior probabilities and ML bootstraps, respectively. Different orders of *Alphaproteobacteria* are labeled by different colors. *Candidatus Pelagibacter ubique* and *Odyssella thessalonicensis* are shown in red.

Mitochondrion-encoded proteins whose phylogeny is closest to the previous *Alphaproteobacteria* phylogenomic tree were selected according to the K tree score method **(** **Table 1** **)** and used to place the mitochondrion within the *Alphaproteobacteria* tree. The nine best protein alignments were concatenated, and ML and Bayesian phylogenies were inferred **(** **Figure 3A** **)**. The Bayesian tree had the same topology as the ML tree, although the Bayesian tree branches were better supported. *O. thessalonicensis* was located alongside the group formed by the *Rhodospirillaceae* and the *Acetobacteraceaea* (Posterior Probability (PP) = 1, BP = 95), and its branch appeared early in the *Alphaproteobacteria* evolution. ML and Bayesian methods showed that *R. americana* mitochondrion grouped with the free-living *Candidatus Pelagibacter ubique* (PP = 0.98, BP = 96), and that together they branched deeply alongside *Rickettsiales*. The mitochondrion phylogenomic tree also suggested an early divergence between *Candidatus Pelagibacter ubique* and the mitochondrion as shown by the length of the branches and the position of the node. Mitochondrion trees without *O. thessalonicensis* presented the same topology for the *Candidatus Pelagibacter ubique* and the mitochondrion, but the branches were not well supported (BP = 63) **(** **Figure 3B** **)**.

**Table 1:** Selection of the nine mitochondrion proteins whose phylogeny is closest to the *Alphaproteobacteria* phylogenomic tree.

K-score	Function
0.48375	LSU ribosomal protein L2p (L8e)
0.51416	NADH-ubiquinone oxidoreductase chain G
0.64953	SSU ribosomal protein S4p (S9e)
0.6564	Cyytochrome c-type biosynthesis protein CcmC
0.68246	LSU tribosomal protein L6p (L9e)
0.68879	NADH-ubiquinone oxidoreductase chain I
0.71266	LSU ribosomal protein L5p (L11e)
071299	Succinate deshydrogenase iron-sulfur protein
0.72572	SSU ribosomal protein S3p (S3e)

## Discussion

Ten years ago, 16S rDNA phylogeny studies described *O. thessalonicensis* as belonging to the *Rickettsiales* order [17]. Here, except from the 16S rDNA tree, we constructed a phylogenomic analysis, more accurate for species delineation [4], and we used the three classic inference methods (ML, MP, NJ), as well as the Bayesian approach, not used in the original paper [17]. The phylogenetic positioning of *O. thessalonicensis* within the *Rickettsiales* clade was only in part verified by the 16S rDNA phylogenetic tree that grouped *O. thessalonicensis* with the *Acetobacteraceae* and as sister taxon of the *Holosporaceae* member, *Caedibacter caryophilus*. In the original paper, the 16S rDNA datasets used were not the same, as the study [17] did not include *Acetobacteraceaea*, *Candidatus Paraholospora*, or *Candidatus Pelagibacter ubique*. Phylogenomic analyses of *Alphaproteobacteria* allowed more robust trees to be built, which help to establish a more reliable position of *O. thessalonicensis* in the *Alphaproteobacteria* family. However, we recognize that the unavailability of the *Holosporaceae* genomes may raise questions on the accuracy of the topology of *O. thessalonicensis*. Nevertheless, the phylogeny of the 65 proteins undoubtedly supports that *Candidatus Pelagibacter ubique* emerged deeply alongside *Rickettsiales*, while the *O. thessalonicensis* branch was well supported between *Rickettsiales* and other *Alphaproteobacteria*. As previously described [2], [13], the selection of the mitochondrion proteins whose phylogeny was closest to the *Alphaproteobacteria* phylogenomic tree was a powerful approach for locating the ancestor of mitochondria. Most studies argued that mitochondria are closely related to the *Rickettsiales* order. However, recent studies on the mitochondrion of the green algae, *C. reinhardtii,* have proposed that most of its mitochondrial protein sister taxon were members of the *Rhizobiales* and the *Rhodobacterales* [11] more often than the *Rickettsiales* order, while a study on *S. cerevisiae* mitochondrion proposes that its sister taxa are more often members of the *Rhizobiales* [16]. The latter study argues the possibility that mitochondrial genomes have a mosaic structure [16], so maybe their origin and evolution is dictated by different elements according to the organism they belong to. Mitochondria are heterogenous and their genomes structure suggests possible genome fusions, addition of different elements and recombination. Different analyses using mitochondria of different organisms would give different results and comparison would not be possible (data not shown). Therefore, for this study, we decided to focus only on the origins of *R. americana* mitochondrion that resembles the most to the proto-mitochondrion ancestor, avoiding to add noise to the phylogenies with the addition of many mitochondria.

It has been proven that adding characters while constructing phylogenetic trees increases the probability that the topology of the obtained tree is correct. The more signals are tested, the more the branches are well supported [21]. *O. thessalonicensis* is, most probably, a non-*Rickettsiales* species the closest related to *Rickettsiales*, as raised by our phylogenomic analyses, it is therefore legitimate to account the *O. thessalonicensis* genome when studying the origin of mitochondria. The addition of new data from *O. thessalonicensis* whole-genome sequencing and the mitochondrial protein selection method using the K tree score partially confirmed the results found by Williams three years ago [2], as well as the ones found by other studies supporting the grouping of the mitochondria with *Alphaproteobacteria* [12], and more specifically with *Rickettsiales* [13] and reinforced the topology presenting *Pelagibacter ubique* in the *Rickettsiales* order [2]. Further and more surprisingly, in our study, the mitochondrion branch emerged as a sister taxon of *Candidatus Pelagibacter ubique*, a result strongly supported by the chosen approach. In the studies mentioned above the mitochondrion branch does not emerge as a sister taxon of *Candidatus Pelagibacter ubique*. Differences are probably due to the different datasets used by each study that do not allow a fair comparison. Moreover, mitochondria seem to have chimeric and heterogenous structures [15] that vary from one organism to another, introducing different results according on which mitochondrion is used in every study. There still is an important debate on whether the free-living organism *Candidatus Pelagibacter ubique* is a member of the *Rickettsiales* order or not, because it is not an intracellular species [15]. Our results link, for the first time undoubtedly, *Candidatus Pelagibacter ubique* to the *Rickettsiales* order and furthermore, with the *Reclimonas americana* mitochondrial ancestor. Trees with or without *O. thessalonicensis* present the same topology, but the positioning of *Candidatus Pelagibacter ubique* as a sister taxon of *Reclinomonas americana* mitochondrion is better supported when *O. thessalonicensis* is used for the tree reconstruction. The node presenting *Candidatus Pelagibacter ubique* and the mitochondrion as sister taxa are better supported when *O. thessalonicensis* is used **(** **Figure 3A,B** **)**. *O. thessalonicensis* data reinforce previous results.

**Figure 4:**
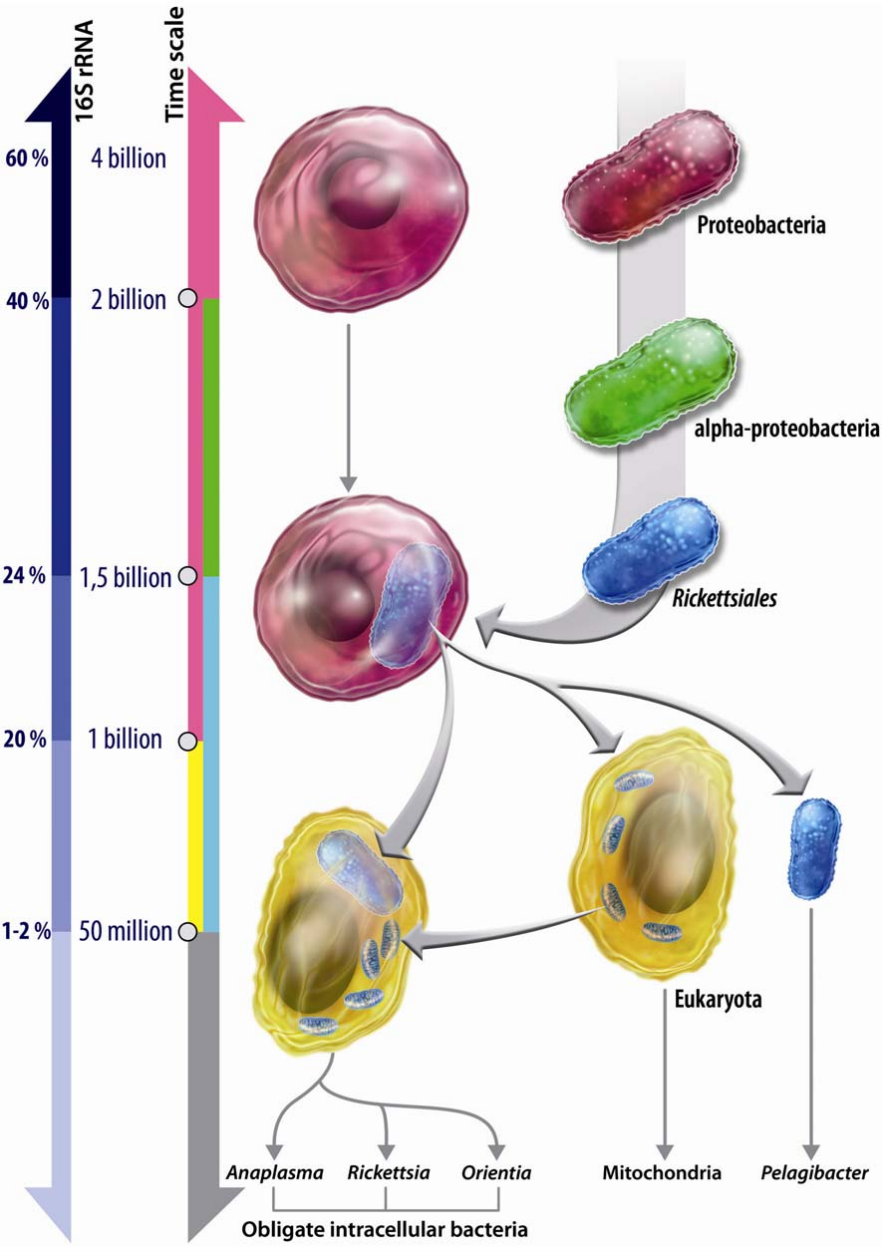
The hypothesis for mitochondrion and free-living *Candidatus Pelagibacter ubique* emergence. From a single facultative intracellular *Rickettsiales* common ancestor, two clades evolved into strict, intracellular species contemporary to the emergence of eukaryotes. *Candidatus Pelagibacter ubique* later evolved into a free-living form. Arrows on the left represent the 16S rRNA percentage divergence scale and the time scale in million of years. A 16S rRNA percentage divergence of 1–2% corresponds to about 50 million years [36]. The arrows on the right represent the emergence events, divergence events and endosymbiotic events.

From the *R. americana* mitochondrion phylogenomic tree, we can suggest two hypotheses. In the first, the *Rickettsiales* (also including *Candidatus Pelagibacter ubique* and the proto-mitochondrion ancestor) had a free-living common ancestor with a rather small genome. There were two endosymbiotic events, one for the *Rickettsiaceae* and one for mitochondria. In the second and more parsimonious hypothesis, there was a single facultative intracellular *Rickettsiales* common ancestor with two clades evolving into a strict intracellular species contemporary to the emergence of eukaryotes and to the creation of proto-mitochondria. In contrast, *Candidatus Pelagibacter ubique* later evolved into a free-living form due to environmental changes that facilitated its adjustment to a relatively stable extracellular environment. *Candidatus Pelagibacter ubique* is the smallest free-living bacterium. Such a massive genome reduction can only be explained by extreme specialisation [22]. However, *Pelagibacter ubique* is a bacterium found in water everywhere in the world. Its small size may therefore witness its previous lifestyle. If its ancestor was a facultative intracellular species the genome reduction took place during its association with a proto-eukaryote **(** **Figure 4** **)**. One single endosymbiotic event is by itself complicated enough and absolutely more parsimonious than two simultaneous endosymbiotic events, so we believe that the most plausible hypothesis is the latter. Moreover, a scenario suggesting that the *Rickettsiales* ancestor became intracellular after diverging from *Candidatus Pelagibacter ubique* would not explain this species extremely small genome. Many hypotheses were described on *Rickettsiales* and mitochondria, in our study we argument on our hypothesis that was never explored before and which was well-supported by our results and by the use of data used for the first time in a study on mitochondrial origins.

The use of new data from *O. thessalonicensis* whole-genome sequencing in the reconstruction of *Alphaproteobacteria* phylogenies, strongly confirmed the emergence of the *R. americana* mitochondrion branch between *Candidatus Pelagibacter ubique* and the other *Rickettsiales* genera. Indeed, the topology of the trees built without *O. thessalonicensis* that presented the *Candidatus Pelagibacter ubique* in the *Rickettsiales* clade was not well supported; therefore, proper determination of its taxonomy was not possible. By adding *O*. *thessalonicensis,* the classification of *Candidatus Pelagibacter ubique* as member of *Rickettsiales* is strongly sustained by all topologies obtained by classic phylogenetic methods, such as ML, and by the Bayesian method. Finally, positioning the *R. americana* mitochondrion ancestor within *Alphaproteobacteria* has proven that the monophyly of *Rickettsiales* and the *R. americana* mitochondrion, and the evolution of *Candidatus Pelagibacter ubique,* emerged from an intracellular to a free-living organism. Currently, the most numerous and successful extracellular bacterial species on Earth, *Candidatus Pelagibacter ubique*, which is highly dominant in both salt and fresh water worldwide [23], is actually a member of the intracellular *Rickettsiales* order.

## Materials and Methods

### Sequence data

*O. thessalonicensis* [Genome Project: ID63085] was isolated from *Acanthamoeba spp.* as described by Britles *et al*. [17]. Genomic DNA was extracted and sequenced using the same method as for *Legionella drancourtii* [24]. The first genome assembly was performed using Newbler software (454 Life Sciences, Roche) and produced 106 contigs (20 scaffolds). Contigs were sent to the RAST platform [25] for rapid gene prediction and annotation. The 16S rDNA sequences from the 49 *Alphaproteobacteria* were extracted from the Ribosomal Database Project [26]. The *Alphaproteobacteria*, *Escherichia coli* K-12 substr. MG1655 [27] and the *Reclinomonas americana* mitochondrion proteomes [28] were downloaded from the NCBI database.

### *Alphaproteobacteria* 16 rDNA phylogeny

The 53 16S rDNA sequences were aligned using MUSCLE [29], and conserved blocks were selected using Gblocks [30]. The curated alignments were realized and used for phylogeny construction. Phylogeny inference was constructed using three different methods, ML, MP, NJ, and a four-category gamma distribution was fit for among-site rate variation. One hundred bootstrap replicates were completed, and the resulting trees were summarized using the majority-rule consensus method. Bootstrap values were considered high when they were higher that 85. Trees were displayed using MEGA4 [31].

### *Alphaproteobacteria* phylogenomics

We used a stringent method in order to establish a protein list that would be representative of all the *Alphaproteobacteria* used in the study. Duplicated genes were discarded from *Alphaproteobacteria* proteomes using the BLASTClust program [32] with a minimum overlap of 70% and a minimum identity of 30%. Proteins considered as non-paralogous were then gathered and used for the cluster of orthologous group (COG) searches. An all-against-all NCBI-BLASTp search was performed on the 42 *Alphaproteobacteria* dataset. All of the proteins present in all species with an identity of 20% and a high-scoring segment pair (HSP) length over 150 amino acids were considered orthologous. Through this method, 65 clusters were identified. Corresponding proteins were aligned with MUSCLE, and conserved blocks were selected with Gblocks. The 65-curated alignments were concatenated and used for phylogeny construction. Phylogenies were constructed using three different methods, ML, MP, NJ, and 100 bootstrap replicates were sampled. *Holosporaceae* were not included because of unavailability of their proteomes. Bootstrap values were considered high when they were higher that 85. The 65 protein sequences from the *Odyssella* were submitted to the GenBank database **(File S1)**.

### Mitochondrion phylogeny relationships

The 67 proteins coded by the mitochondrial DNA of *Reclinomonas americana* were compared to the *Alphaproteobacteria* proteomes using NCBI4 BLASTp. Mitochondrial proteins with the best blast hits (BBHs) and an e-value under e^-20^ were selected; 59 proteins matched these criteria. For each of the 59 successful proteins, corresponding BBHs were aligned, and an ML tree was built using PhyML [33]. Trees with 42 leaves were compared to the *Alphaproteobacteria* multiprotein tree using the K tree score. Only 43 trees had 42 leaves. The nine best trees were determined according to the K tree score [34]. Mitochondrion-encoded proteins were added to each of the nine successful alignments. The conserved blocks were concatenated in a single 728-amino acid alignment, and the mitochondrion phylogeny was inferred by ML and Bayesian inference methods. For the Bayesian approach, phylogeny was performed using MrBayes [35]; the GTR matrix was used, and model parameters (gamma shape and proportion of invariant) were allowed to vary through the Markov Chain Monte Carlo Chain (MCMC). Four MCMC chains were run for one million generations and sampled every 100th generation. The first 100,000 trees were discarded, and the “sumt” command of MrBayes was used to compute the clade posterior probabilities. *Holosporaceae* were not included because of unavailability of their proteomes. Bootstrap values were considered high when they were higher that 85 and PP higher that 0.85.The trees were rendered with MEGA4.

## Supporting Information

File S1 **65** ***Odyssella thessalonicensis*** **proteins.** http://www.biomedcentral.com/imedia/1660338084382525/supp1.txt
(TXT)
